# Standardised inventories of lepidopterans and odonates from Serra da Estrela Natural Park (Portugal) - setting the scene for mountain biodiversity monitoring

**DOI:** 10.3897/BDJ.11.e99558

**Published:** 2023-03-15

**Authors:** Mário Boieiro, Sandra Antunes, Hugo Figueiredo, Albano Soares, Ana Lopes, Eva Monteiro, Patrícia Garcia-Pereira, Carla Rego, José Conde, Paulo A.V. Borges, Artur R.M. Serrano

**Affiliations:** 1 Centre for Ecology, Evolution and Environmental Changes (cE3c)/Azorean Biodiversity Group, CHANGE – Global Change and Sustainability Institute, Faculty of Agricultural Sciences and Environment, University of the Azores, Angra do Heroísmo, Azores, Portugal Centre for Ecology, Evolution and Environmental Changes (cE3c)/Azorean Biodiversity Group, CHANGE – Global Change and Sustainability Institute, Faculty of Agricultural Sciences and Environment, University of the Azores Angra do Heroísmo, Azores Portugal; 2 TAGIS - Centro de Conservação das Borboletas de Portugal, Avis, Portugal TAGIS - Centro de Conservação das Borboletas de Portugal Avis Portugal; 3 CISE - Centro de Interpretação da Serra da Estrela, Município de Seia, Seia, Portugal CISE - Centro de Interpretação da Serra da Estrela, Município de Seia Seia Portugal; 4 Departamento de Biologia, Universidade de Aveiro, Aveiro, Portugal Departamento de Biologia, Universidade de Aveiro Aveiro Portugal; 5 Centre for Ecology, Evolution and Environmental Changes (cE3c) & CHANGE – Global Change and Sustainability Institute, Faculty of Sciences, University of Lisbon, Lisboa, Portugal Centre for Ecology, Evolution and Environmental Changes (cE3c) & CHANGE – Global Change and Sustainability Institute, Faculty of Sciences, University of Lisbon Lisboa Portugal

**Keywords:** butterflies, Lepidoptera, damselflies, dragonflies, Odonata, elevation gradient, mountain lakes, protected areas, Habitats Directive

## Abstract

**Background:**

Mountain insect biodiversity is unique, but is menaced by different drivers, particularly climate and land-use changes. In mainland Portugal, the highest mountain - Serra da Estrela - is one of the most important biodiversity hotspots, being classified as Natural Park since 1976. Many lepidopteran and odonate species, including rare and protected species, are known to occur in Serra da Estrela, but basic knowledge on their abundance, distribution and ecology is still lacking. Standardised sampling of these communities is crucial to provide valuable biological information to support short-term decision-making for conservation management, setting simultaneously the standards for mountain biodiversity monitoring aiming to tackle the effects of environmental change in the long-term.

**New information:**

This study reports novel information on lepidopteran and odonate species diversity, distribution and abundance from Serra da Estrela Natural Park (Portugal). Seventy-two lepidopteran and 26 odonate species were sampled in this protected area, including the first findings of *Apaturailia* (Denis & Schiffermüller, 1775), *Macromiasplendens* (Pictet, 1843) and *Vanessavirginiensis* (Drury, 1773). New populations of *Euphydriasaurinia* (Rottemburg, 1775) and *Oxygastracurtisii* (Dale, 1834), protected species under the Habitats Directive, were found in this Natural Park and novel distribution and ecological data were collected for most species, including several rare species and subspecies [e.g. *Aeshnajuncea* (Linnaeus, 1758), *Coenonymphaglycerioniphioides* Staudinger, 1870, *Cyanirissemiargus* (Rottemburg, 1775) and *Sympetrumflaveolum* (Linnaeus, 1758)]. All data were collected using standardised sampling allowing its use as a baseline for biodiversity monitoring in Serra da Estrela.

## Introduction

Mountain ecosystems are crucial for global biodiversity conservation since they host high numbers of plant and animal species, including many rare, endemic and those of conservation concern (Hodkinson and Jackson 2005). During the last few decades, research and monitoring of mountain biodiversity has been key to unveiling the drivers of species diversity and community composition and to provide scientifically-supported guidance to manage these unique and fragile ecosystems (Nogués-Bravo et al. 2008, Ashton et al. 2011). For instance, the Global Mountain Biodiversity Assessment (GMBA) initiative has been key on the assessment, conservation and sustainable management of mountain biodiversity (see Payne et al. (2017)). This is extremely important as mountain ecosystems worldwide are menaced by various threats, such as global warming, species introductions, vegetation/land-use changes and water extraction, that often act synergistically (Schmeller 2022). Climate change is considered one of the most impactful threats to mountain biodiversity with many reports stating changes in species composition of mountain communities, upslope shifts of species ranges and even local extinctions in response to temperature increases (Sekercioglu et al. 2008, Chen 2009, Lenoir and Svenning 2015). In addition, human activities have historically played (and still play) an important role as drivers of biodiversity patterns in mountain ecosystems; often mountains supported intensive and/or extensive agricultural, forestry and livestock production practices, were subjected to frequent fires, water extraction and recreational activities. The biodiversity of Iberian mountains, including Serra da Estrela, face this same kind of threats (Wilson et al. 2007, Múrria 2020) and, due to the vulnerability of these ecosystems to environmental change, is key for implementing long-term biodiversity monitoring programmes to tackle changes in abiotic and biotic conditions and supporting decision-making for conservation management. Several invertebrate groups have been the target of biodiversity monitoring programmes in mountain ecosystems worldwide since they provide valuable information on the state of the environment in an effective and efficient way. Lepidopterans and (to a less extent) odonates are two invertebrate groups often selected in biodiversity monitoring since they have a well-known ecology and taxonomy, and are cost-effective to survey (Oertli 2008, Kessler 2011, Acquah-Lamptey et al. 2013, Gerlach et al. 2013, Zografou 2014). In this study, we provide novel information on lepidopteran and odonate species diversity, distribution and abundance from representative habitats of Serra da Estrela Natural Park (Portugal) following a standardised sampling protocol, aiming to set a reference work for biodiversity monitoring in this emblematic protected area.

## General description

### Purpose

We present new taxonomic, distribution and abundance data on the lepidopterans and odonates of Serra da Estrela Natural Park following an extensive survey of adult forms using standardised sampling. The data encompass the elevation gradient of Serra da Estrela, the highest mountain in mainland Portugal and includes information from a variety of habitat types (mountain streams, mountain lakes and montane vegetation) during two consecutive years (2013 and 2014).

## Project description

### Title

Biodiversity, endemic and protected species associated with mountain lakes and streams of Serra da Estrela

### Personnel

Mário Boieiro, José Conde and Artur Serrano planned the project and designed the sampling strategy; Sandra Antunes, Albano Soares, Hugo Figueiredo, Ana Lopes, Eva Monteiro, Patrícia Garcia-Pereira, Carla Rego, José Conde and Mário Boieiro participated in fieldwork. Mário Boieiro and Paulo Borges performed the biodiversity data curation in Darwin Core format.

### Study area description

The study took place in Serra da Estrela, the highest mountain in continental Portugal (with 1993 m). Serra da Estrela includes the western extreme of the Iberian Central System which is considered one of the main mountain systems in the Iberian Peninsula. Serra da Estrela is classified as Natural Park since 1976, is part of the Natura 2000 network and its upper areas are included in the Ramsar Convention (ICNF 2022). All study sites are included in the Serra da Estrela Natural Park, encompassing a considerable elevation gradient and habitat diversity. The study area is characterised by Atlantic and Mediterranean climates and different biogeographic regions, being an important area for biodiversity conservation, particularly for montane species (ICNF 2022).

### Design description

Sampling of lepidopterans and odonates took place in three main habitat types, namely mountain streams, mountain lakes and montane vegetation (Fig. 1; Table 1). We sampled the margins of three mountain streams (Fervença, Caniça and Loriga) at three elevation levels (approximately 500, 1000 and 1500m) (Fig. 1a) and 18 mountain lakes (including both natural and artificial lakes), most of them being located in the Central Plateau of Serra da Estrela (Fig. 1b). Lepidopterans and odonates were also sampled from 12 sites of representative montane vegetation of Serra da Estrela, including *Juniperus*-, *Erica*- and *Genista*-dominated scrublands and *Nardus*-dominated grasslands (Fig. 1c). Overall, 39 sites were sampled during this study (Table 1).

### Funding

This work was financed by the Energias de Portugal (EDP) Fund for Biodiversity 2011 through project "Biodiversidade, endemismos e espécies protegidas associadas às lagoas e cursos de água da Serra da Estrela: valorização de um século de aproveitamento hidroeléctrico". Fundação para a Ciência e a Tecnologia funded APC through project UIDB/00329/2020–2024 and supported MB by contract DL57/2016/CP1375/CT0001.

## Sampling methods

### Study extent

The study was carried out in Serra da Estrela Natural Park encompassing the elevation gradient and the diversity of habitats of this protected area.

### Sampling description

Lepidopterans and odonates were sampled using a standardised methodology to ensure the possibility of biodiversity data comparison between study sites and to set a reference for mountain biodiversity monitoring in Serra da Estrela Natural Park. Insect sampling followed the Pollard and Yates methodology (Pollard and Yates 1993): a 150 m linear transect was set in each study site and adult insects of the target groups were recorded when observed at a distance of up to 5 m ahead of the researcher and 2.5 m from each side. The insects were captured with the help of a sweeping net only in case of need to confirm their species identity, being immediately released afterwards. Sampling was carried out between 10 am and 6 pm and under favourable climatic conditions (i.e. sampling was not performed under rainy, windy, cloudy and hot weather conditions). The data were collected during the seasonal peak of activity of adult lepidopterans and odonates in Serra da Estrela in two consecutive years (2013 and 2014).

### Quality control

Lepidopterans and odonates were identified by trained taxonomists (Albano Soares, Hugo Figueiredo and Sandra Antunes) during fieldwork.

## Geographic coverage

### Description

Serra da Estrela Natural Park, Portugal

### Coordinates

 and Latitude Latitude; and Longitude: -7.886433ºW to -7.200313 Longitude.

## Taxonomic coverage

### Taxa included

**Table:** 

Rank	Scientific Name	Common Name
order	Odonata	odonates; dragonflies and damselflies
order	Lepidoptera	lepidopterans; butterflies

## Temporal coverage

### Notes

The data were collected during the seasonal peak of activity of adult lepidopterans and odonates in Serra da Estrela, which lasts from late spring to late summer. Data were collected during two consecutive years: from June to September 2013 and from July to September 2014.

## Usage licence

### Usage licence

Creative Commons Public Domain Waiver (CC-Zero)

## Data resources

### Data package title

Invertebrate biodiversity of Serra da Estrela Natural Park - EDP Biodiversity Fund

### Number of data sets

4

### Data set 1.

#### Data set name

Standardised sampling of lepidopterans (Lepidoptera) in Serra da Estrela (Portugal) - 2013 and 2014 - Event Table

#### Data format

Darwin Core

#### Character set

UTF-8

#### Download URL

http://ipt.gbif.pt/ipt/resource?r=lepidoptera_estrela

#### Data format version

1.6

#### Description

The dataset was published in the Global Biodiversity Information Facility platform, GBIF (Boieiro 2022a). The following data table includes all the records for which a taxonomic identification of the species was possible. The dataset submitted to GBIF is structured as a sample event dataset that has been published as a Darwin Core Archive (DwCA), which is a standardised format for sharing biodiversity data as a set of one or more data tables. The core data file contains 245 records (eventID). This IPT (Integrated Publishing Toolkit) archives the data and thus serves as the data repository. The data and resource metadata are available for download in the Portuguese GBIF Portal IPT (Boieiro 2022a).

**Data set 1.** 

Column label	Column description
id	Unique identification code for sampling event data.
eventID	Identifier of the events, unique for the dataset.
samplingProtocol	The sampling protocol used to capture the species.
sampleSizeValue	The numeric amount of time spent in each sampling.
sampleSizeUnit	The unit of the sample size value.
samplingEffort	The amount of effort expended during an Event.
eventDate	Date or date range the record was collected.
year	The four-digit year in which the Event occurred, according to the Common Era Calendar.
month	The integer month in which the Event occurred.
day	The integer day of the month on which the Event occurred.
habitat	The habitat from which the sample was obtained.
locationID	Identifier of the location.
country	Country of the sampling site (in this case, Portugal).
countryCode	ISO code of the country of the sampling site (PT - Portugal).
municipality	Municipality of the sampling site.
locality	Name of the locality.
minimumElevationInMetres	The lower limit of the range of elevation (altitude, usually above sea level), in metres.
decimalLatitude	Approximate centre point decimal latitude of the field site in GPS coordinates.
decimalLongitude	Approximate centre point decimal longitude of the field site in GPS coordinates.
geodeticDatum	The ellipsoid, geodetic datum or spatial reference system (SRS) upon which the geographic coordinates given in decimalLatitude and decimalLongitude are based.
coordinateUncertaintyInMetres	Uncertainty of the coordinates of the centre of the sampling plot, in metres.
coordinatePrecision	Precision of the coordinates.
georeferenceSources	A list (concatenated and separated) of maps, gazetteers or other resources used to georeference the Location, described specifically enough to allow anyone in the future to use the same resources.
verbatimLatitude	The verbatim original latitude of the Location.
verbatimLongitude	The verbatim original longitude of the Location.
verbatimSRS	The ellipsoid, geodetic datum or spatial reference system (SRS) upon which coordinates given in verbatimLatitude and verbatimLongitude or verbatimCoordinates are based.

### Data set 2.

#### Data set name

Standardised sampling of lepidopterans (Lepidoptera) in Serra da Estrela (Portugal) - 2013 and 2014 - Occurrence Table

#### Data format

Darwin Core

#### Character set

UTF

#### Download URL

http://ipt.gbif.pt/ipt/resource?r=lepidoptera_estrela

#### Data format version

1.6

#### Description

The dataset was published in the Global Biodiversity Information Facility platform, GBIF (Boieiro 2022a). The following data table includes all the records for which a taxonomic identification of the species was possible. The dataset submitted to GBIF is structured as an occurrence table that has been published as a Darwin Core Archive (DwCA), which is a standardised format for sharing biodiversity data as a set of one or more data tables. The core data file contains 1614 records (occurrenceID). This IPT (Integrated Publishing Toolkit) archives the data and thus serves as the data repository. The data and resource metadata are available for download in the Portuguese GBIF Portal IPT (Boieiro 2022a).

**Data set 2.** 

Column label	Column description
id	Unique identification code for species abundance data. Equivalent here to eventID.
type	The nature or genre of the resource, as defined by the Darwin Core standard.
licence	Reference to the licence under which the record is published (CC-BY) 4.0.
institutionID	The identity of the institution publishing the data.
institutionCode	The identity of the collection publishing the data.
basisOfRecord	The nature of the data record.
occurrenceID	Identifier of the record, coded as a global unique identifier.
recordedBy	A list (concatenated and separated) of names of people, groups or organisations who performed the sampling in the field.
organismQuantity	A number or enumeration value for the quantity of organisms.
organismQuantityType	The type of quantification system used for the quantity of organisms.
lifeStage	The life stage of the organisms captured.
establishmentMeans	The process of establishment of the species in the location, using a controlled vocabulary: 'native', 'introduced', 'endemic', "unknown".
eventID	Identifier of the events, unique for the dataset.
identifiedBy	A list (concatenated and separated) of names of people, groups or organisations who assigned the Taxon to the subject.
dateIdentified	The date on which the subject was determined as representing the Taxon.
scientificName	Complete scientific name including author and year.
kingdom	Kingdom name.
phylum	Phylum name.
class	Class name.
order	Order name.
family	Family name.
genus	Genus name.
specificEpithet	Specific epithet.
infraspecificEpithet	Subspecies epithet.
taxonRank	Lowest taxonomic rank of the record.
scientificNameAuthorship	Name of the author of the lowest taxon rank included in the record.

### Data set 3.

#### Data set name

Standardised sampling of odonates (Odonata) in Serra da Estrela (Portugal) - 2013 and 2014 - Event Table

#### Data format

Darwin Core

#### Character set

UTF-8

#### Download URL

http://ipt.gbif.pt/ipt/resource?r=odonata_estrela_portugal

#### Data format version

1.6

#### Description

The dataset was published in the Global Biodiversity Information Facility platform, GBIF (Boieiro 2022b). The following data table includes all the records for which a taxonomic identification of the species was possible. The dataset submitted to GBIF is structured as a sample event dataset that has been published as a Darwin Core Archive (DwCA), which is a standardised format for sharing biodiversity data as a set of one or more data tables. The core data file contains 172 records (eventID). This IPT (Integrated Publishing Toolkit) archives the data and thus serves as the data repository. The data and resource metadata are available for download in the Portuguese GBIF Portal IPT (Boieiro 2022b).

**Data set 3.** 

Column label	Column description
id	Unique identification code for sampling event data.
eventID	Identifier of the events, unique for the dataset.
samplingProtocol	The sampling protocol used to capture the species.
sampleSizeValue	The numeric amount of time spent in each sampling.
sampleSizeUnit	The unit of the sample size value.
samplingEffort	The amount of effort expended during an Event.
eventDate	Date or date range the record was collected.
year	The four-digit year in which the Event occurred, according to the Common Era Calendar.
month	The integer month in which the Event occurred.
day	The integer day of the month on which the Event occurred.
habitat	The habitat from which the sample was obtained.
locationID	Identifier of the location.
country	Country of the sampling site (in this case Portugal).
countryCode	ISO code of the country of the sampling site.
municipality	Municipality of the sampling site.
locality	Name of the locality.
minimumElevationInMetres	The lower limit of the range of elevation (altitude, usually above sea level), in metres.
decimalLatitude	Approximate centre point decimal latitude of the field site in GPS coordinates.
decimalLongitude	Approximate centre point decimal longitude of the field site in GPS coordinates.
geodeticDatum	The ellipsoid, geodetic datum or spatial reference system (SRS) upon which the geographic coordinates given in decimalLatitude and decimalLongitude are based.
coordinateUncertaintyInMeters	Uncertainty of the coordinates of the centre of the sampling plot, in metres.
coordinatePrecision	Precision of the coordinates.
georeferenceSources	A list (concatenated and separated) of maps, gazetteers or other resources used to georeference the Location, described specifically enough to allow anyone in the future to use the same resources.
verbatimLatitude	The verbatim original latitude of the Location.
verbatimLongitude	The verbatim original longitude of the Location.
verbatimSRS	The ellipsoid, geodetic datum or spatial reference system (SRS) upon which coordinates given in verbatimLatitude and verbatimLongitude or verbatimCoordinates are based.

### Data set 4.

#### Data set name

Standardised sampling of odonates (Odonata) in Serra da Estrela (Portugal) - 2013 and 2014 - Occurrence Table

#### Data format

Darwin Core

#### Character set

UTF-8

#### Download URL

http://ipt.gbif.pt/ipt/resource?r=odonata_estrela_portugal

#### Data format version

1.6

#### Description

The dataset was published in the Global Biodiversity Information Facility platform, GBIF (Boieiro 2022b). The following data table includes all the records for which a taxonomic identification of the species was possible. The dataset submitted to GBIF is structured as an occurrence table that has been published as a Darwin Core Archive (DwCA), which is a standardised format for sharing biodiversity data as a set of one or more data tables. The core data file contains 520 records (occurrenceID). This IPT (Integrated Publishing Toolkit) archives the data and thus serves as the data repository. The data and resource metadata are available for download in the Portuguese GBIF Portal IPT (Boieiro 2022b).

**Data set 4.** 

Column label	Column description
id	Unique identification code for species abundance data. Equivalent here to eventID.
type	The nature or genre of the resource, as defined by the Darwin Core standard.
licence	Reference to the licence under which the record is published (CC-BY) 4.0.
institutionID	The identity of the institution publishing the data.
institutionCode	The identity of the collection publishing the data.
basisOfRecord	The nature of the data record.
occurrenceID	Identifier of the record, coded as a global unique identifier.
recordedBy	A list (concatenated and separated) of names of people, groups or organisations who performed the sampling in the field.
organismQuantity	A number or enumeration value for the quantity of organisms.
organismQuantityType	The type of quantification system used for the quantity of organisms.
lifeStage	The life stage of the organisms captured.
establishmentMeans	The process of establishment of the species in the location, using a controlled vocabulary: 'native', 'introduced', 'endemic', "unknown".
eventID	Identifier of the events, unique for the dataset.
identifiedBy	A list (concatenated and separated) of names of people, groups or organisations who assigned the Taxon to the subject.
dateIdentified	The date on which the subject was determined as representing the Taxon.
scientificName	Complete scientific name including author and year.
kingdom	Kingdom name.
phylum	Phylum name.
class	Class name.
order	Order name.
family	Family name.
genus	Genus name.
specificEpithet	Specific epithet.
taxonRank	Lowest taxonomic rank of the record.
scientificNameAuthorship	Name of the author of the lowest taxon rank included in the record.

## Additional information

### Results

During the two-year study, we observed 7339 adult insects from 98 species of the two target groups (Lepidoptera and Odonata) in Serra da Estrela Natural Park. Overall, we identified 72 lepidopteran species (3971 observed individuals) from five different families (Table 2) and 26 odonate species (3368 observed individuals) from ten different families (Table 3), being 11 zygopterans and the remaining 15 anisopterans. We report for the first time the finding of *Apaturailia*, *Macromiasplendens* and *Vanessavirginiensis* in this protected area, jointly with the location of new populations of the Habitats Directive protected species *Euphydriasaurinia* and *Oxygastracurtisii* (Fig. 2a).

*Coenonymphaglycerioniphioides* and *Cyanirissemiargus*, both considered threatened by extinction in Portugal (Maravalhas 2003), occurred in several study sites in Serra da Estrela (Fig. 2b). *Aeshnajuncea* and *Sympetrumflaveolum*, two narrow-range odonate species in Portugal and both classified as threatened (Maravalhas and Soares 2013), were found in several sites, mostly located in the Central Plateau of Serra da Estrela (Fig. 2c, d). Several lepidopteran and odonate species (including the newly-recorded species *Apaturailia*, *Macromiasplendens* and *Vanessavirginiensis*) were found to be rare in the study area, occurring in low abundance in just a few sites.

The species abundance distributions of the two study groups show a bimodal pattern with high number of species in moderately low and moderately high abundance classes; fewer species were found to be low- or high-abundant (Fig. 3a). Five lepidopterans, including the montane *Satyrusactaea* (Esper, 1781), occurred in high abundance (> 256 individuals) in the study area and two odonates [*Enallagmacyathigerum* (Charpentier, 1840) and *Libellulaquadrimaculata* Linnaeus, 1758] were also very abundant (> 512 individuals). The occupancy frequency distributions of the two study groups followed a common pattern: most species were found in a few number of sites and a scarce number of species was widespread (Fig. 3b). Only two lepidopterans, *Coliascroceus* (Geoffroy, 1785) and *Pierisrapae* (Linnaeus, 1758) were found in most study sites (respectively 38 and 36, out of 39).

**Figure 4.**
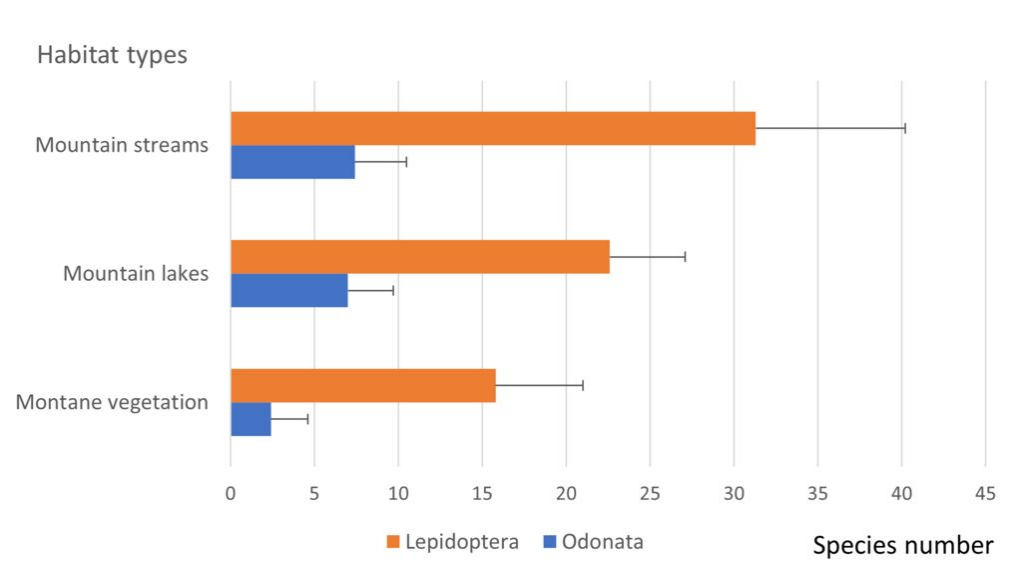
Average number (+SD) of lepidopteran and odonate species found in sites of the three main habitat types.

Average values of lepidopteran and odonate species richness were higher in mountain streams and lakes (Fig. 4). Fewer species were detected in sites in montane vegetation.

### Discussion

The diversity of lepidopteran and odonate species in Serra da Estrela is one of the highest in Portugal since the elevation gradient of this mountain determines marked differences in abiotic and biotic conditions at relatively short distances, thus driving habitat diversity, species distributions and spatial patterns of biodiversity (Ferreira et al. 2009). Additionally, due to its geophysical characteristics, Serra da Estrela has several montane habitat types that are exclusive or poorly represented in the country (Jansen 2002), where we can find range-restricted species, such as *Aeshnajuncea*, *Sympetrumflaveolum* and *Satyrusactaea* (Maravalhas 2003, Maravalhas and Soares 2013).

During the two-year study, we sampled the lepidopteran and odonate communities from 39 sites in Serra da Estrela Natural Park, improving the species inventory by recording three new species to this protected area and collecting novel data on species abundance, distribution and ecology for nearly one hundred species, including two protected under the Habitats Directive. Interestingly, a number of lepidopteran and odonate species were found in low abundance in a few number of sites (Tables 2, 3; Fig. 3), suggesting the need to further investigate the distribution and population size of these species in Serra da Estrela. Despite the occurrence of several odonate and lepidopteran species of conservation concern in Serra da Estrela (both listed in the Habitats Directive or classified as threatened by national experts, see Maravalhas (2003), Maravalhas and Soares (2013)), we are unaware of studies targeting the collection of crucial baseline data on species abundance, distribution and threats that are much needed to support their conservation in this Natural Park. In addition, it will be important to sample a higher number of sites and habitats in this large protected area (with nearly 90,000 ha) to ascertain the rarity of several species that may in fact be undersampled. The combined analysis of species abundance distributions and occupancy frequency distributions also highlighted a few species that are relatively abundant and widespread in Serra da Estrela, like the odonates *Enallagmacyathigerum* and *Libellulaquadrimaculata* and the lepidopterans *Coliascroceus* and *Pierisrapae*. These species are common in the country (Maravalhas 2003, Maravalhas and Soares 2013) and seem to cope with the wide variety of ecological conditions through the elevation gradient of Serra da Estrela. Not surprisingly, we found a higher average number of odonate and lepidopteran species in mountain streams and lakes than in montane vegetation. Odonates depend on these aquatic ecosystems for reproduction and larvae development, while lepidopterans seem to benefit from a larger diversity of host plants.

Our study presents baseline information on species abundance and distribution following standardised sampling in representative habitats of Serra da Estrela, aiming to set a reference for long-term monitoring of biodiversity in this mountain. The biodiversity of Serra da Estrela faces several threats, particularly due to climate change, wildfires, the spread of invasive species and excessive water extraction, all of which are crucial for assessing their impact on montane plant and animal communities. Both odonates and lepidopterans are important bioindicators and many studies showed that they may provide valuable information as global change sentinels since their populations respond quickly to environmental change and at very fine scales (Hassall 2015, Hill et al. 2021).

## Figures and Tables

**Figure 1a.**
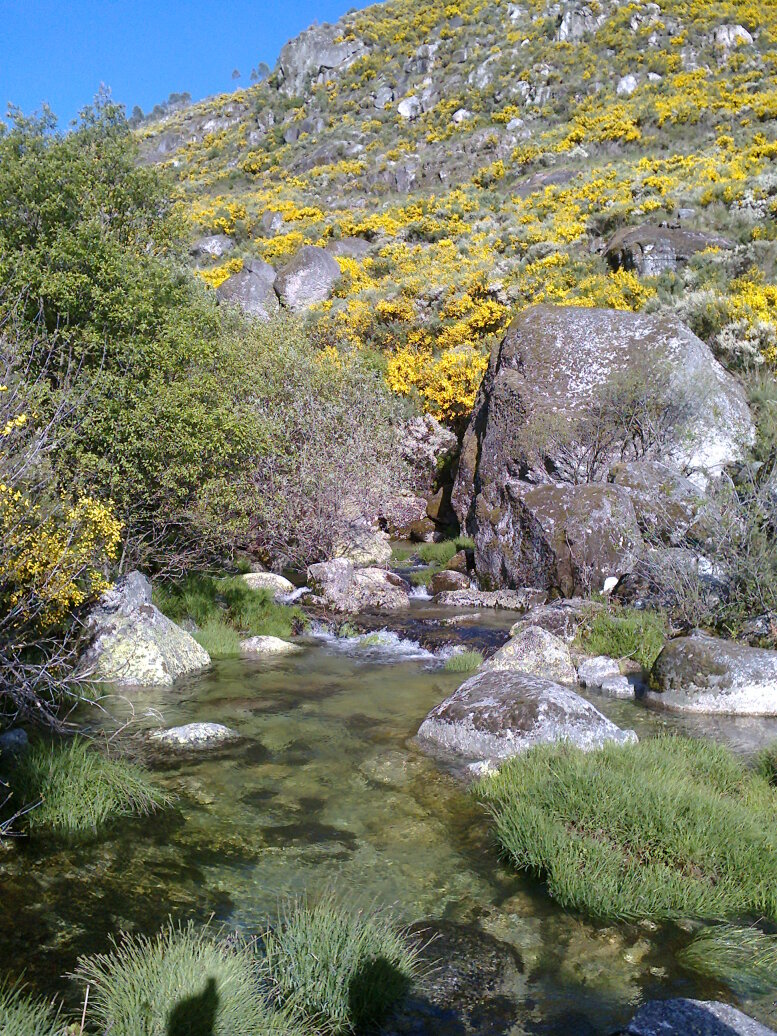
Mountain stream (Ribeira de Loriga) in Serra da Estrela (photo by Mário Boieiro).

**Figure 1b.**
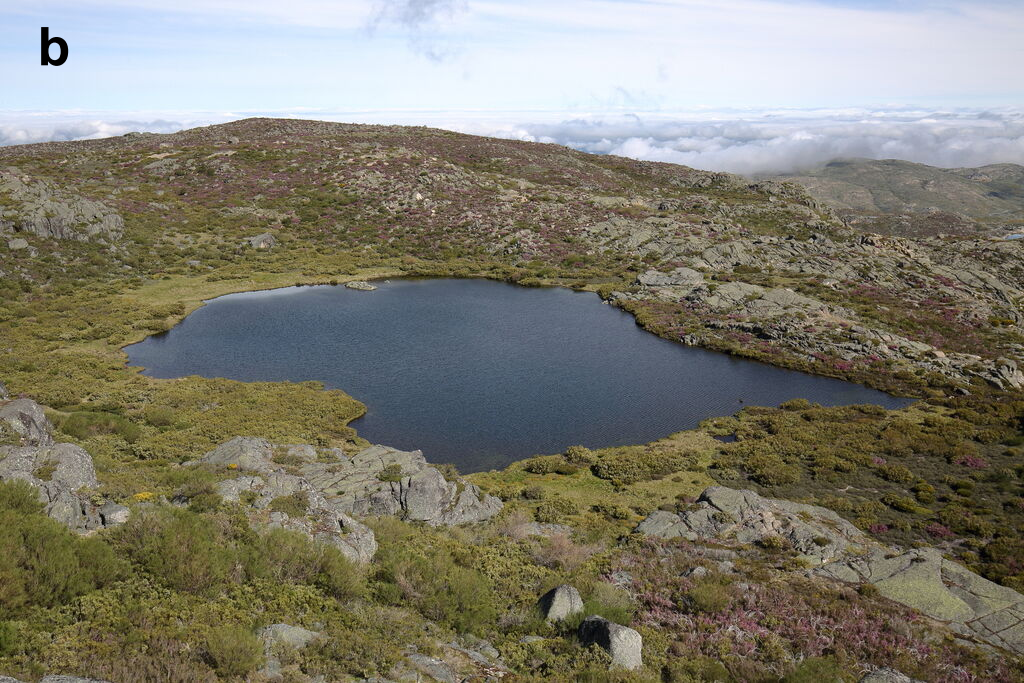
Mountain lake (Lagoa Redonda) in Serra da Estrela (photo by José Conde).

**Figure 1c.**
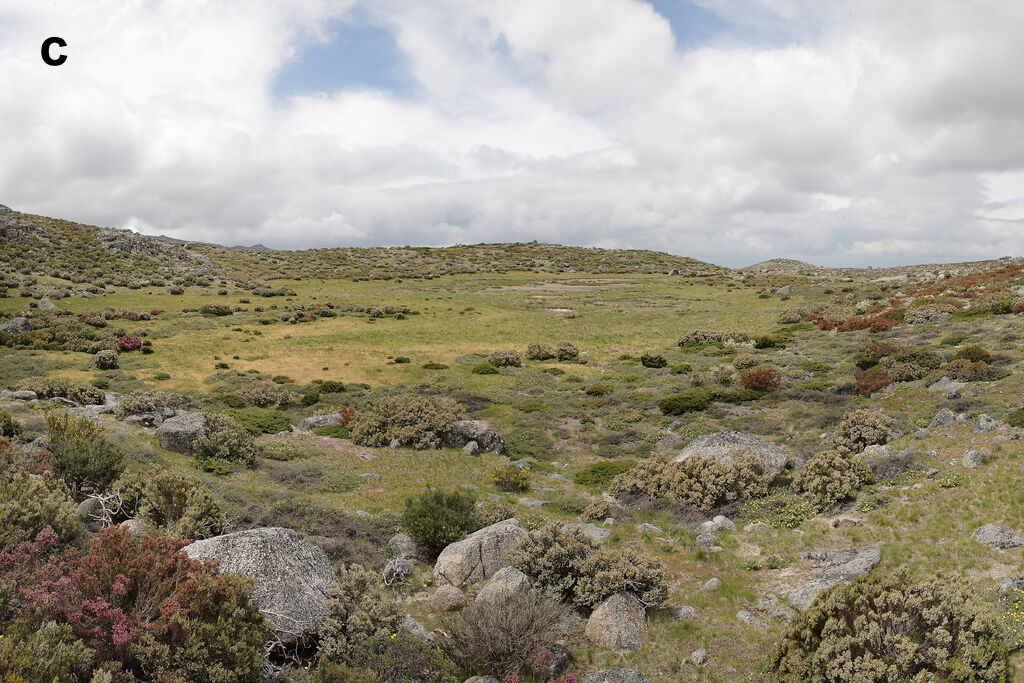
Montane vegetation (near Lagoa Seca) in Serra da Estrela (photo by José Conde).

**Figure 2a.**
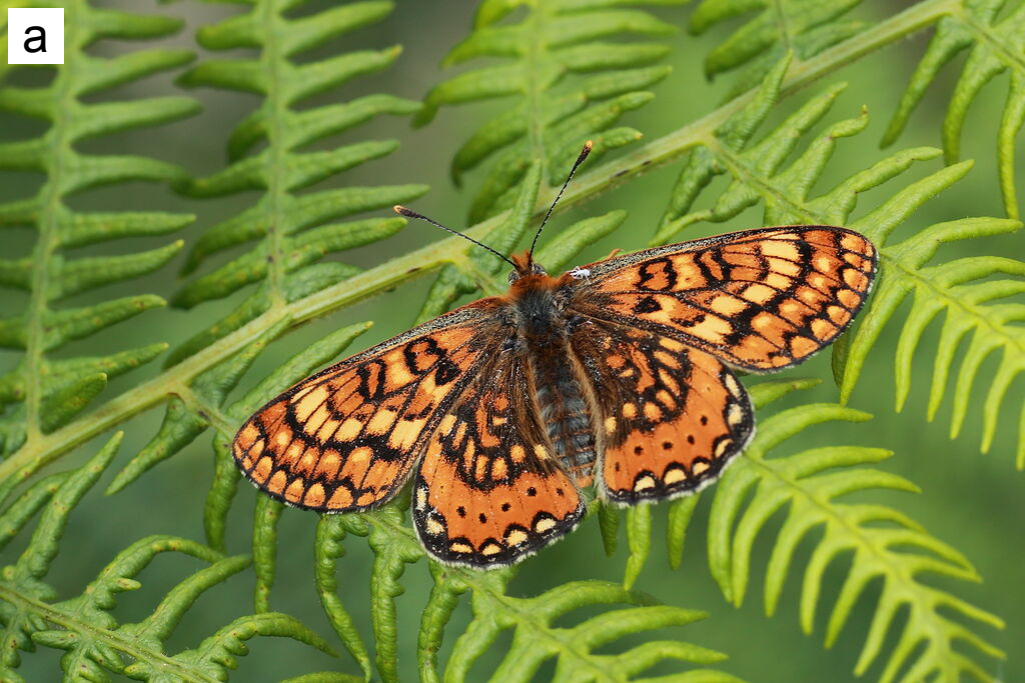
The protected butterfly *Euphydriasaurinia* (photo by José Conde).

**Figure 2b.**
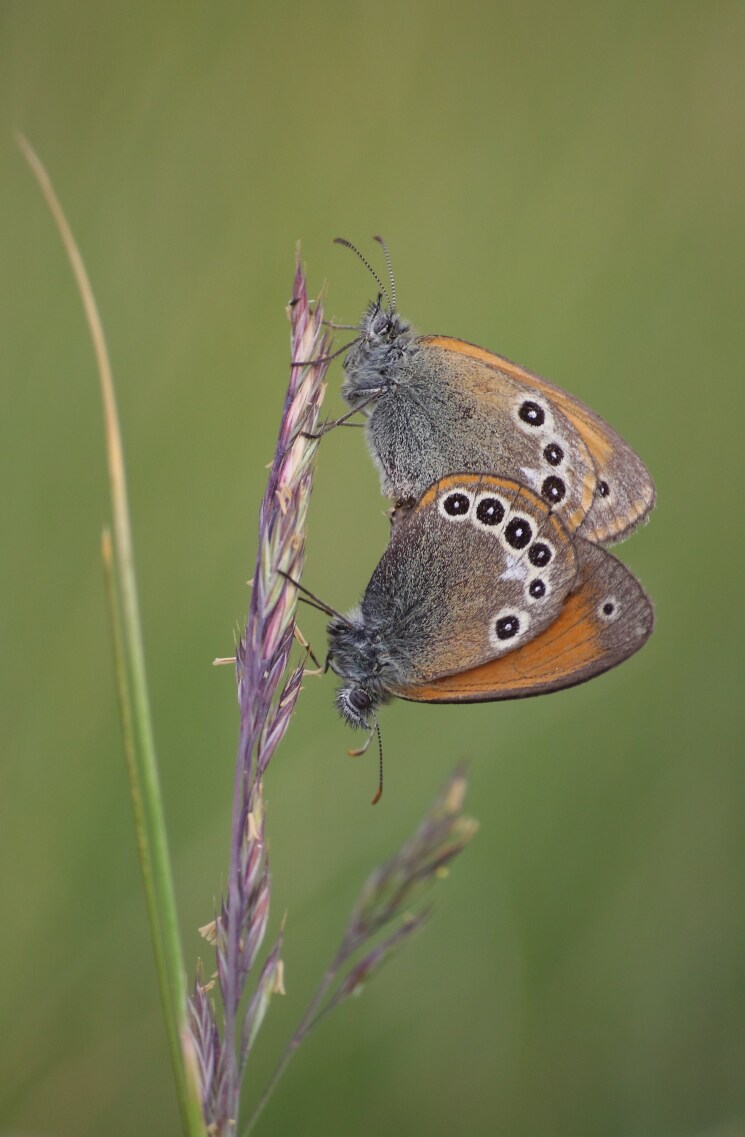
The butterfly *Coenonymphaglycerioniphioides* (photo by Albano Soares).

**Figure 2c.**
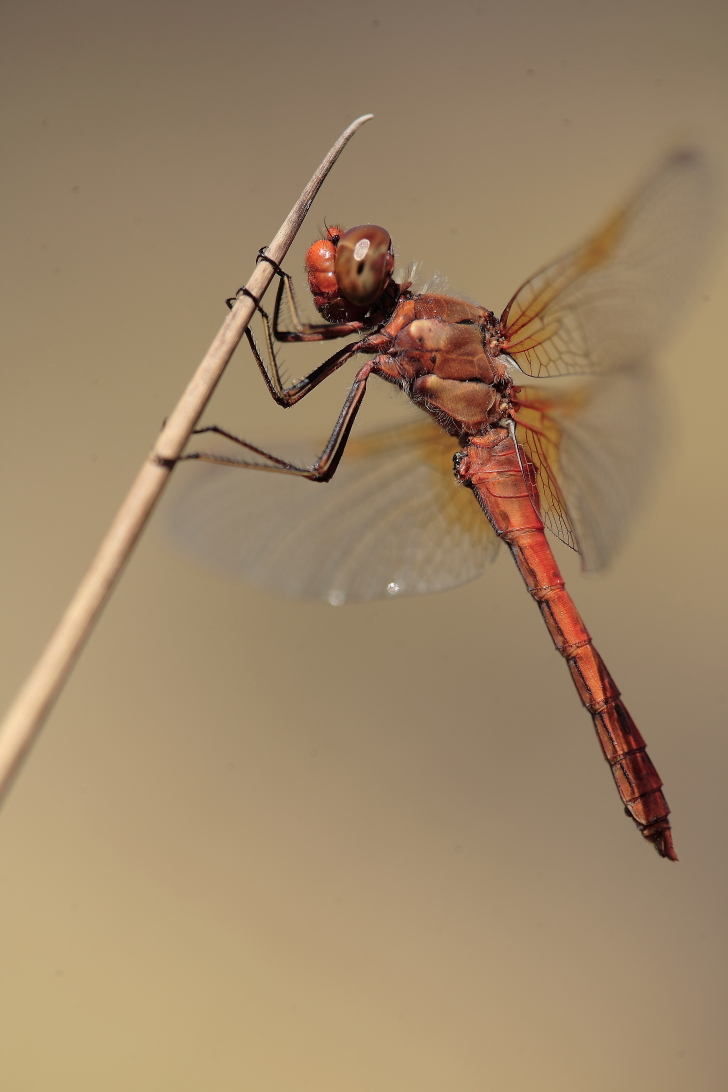
The dragonfly *Sympetrumflaveolum* (photo by José Conde).

**Figure 2d.**
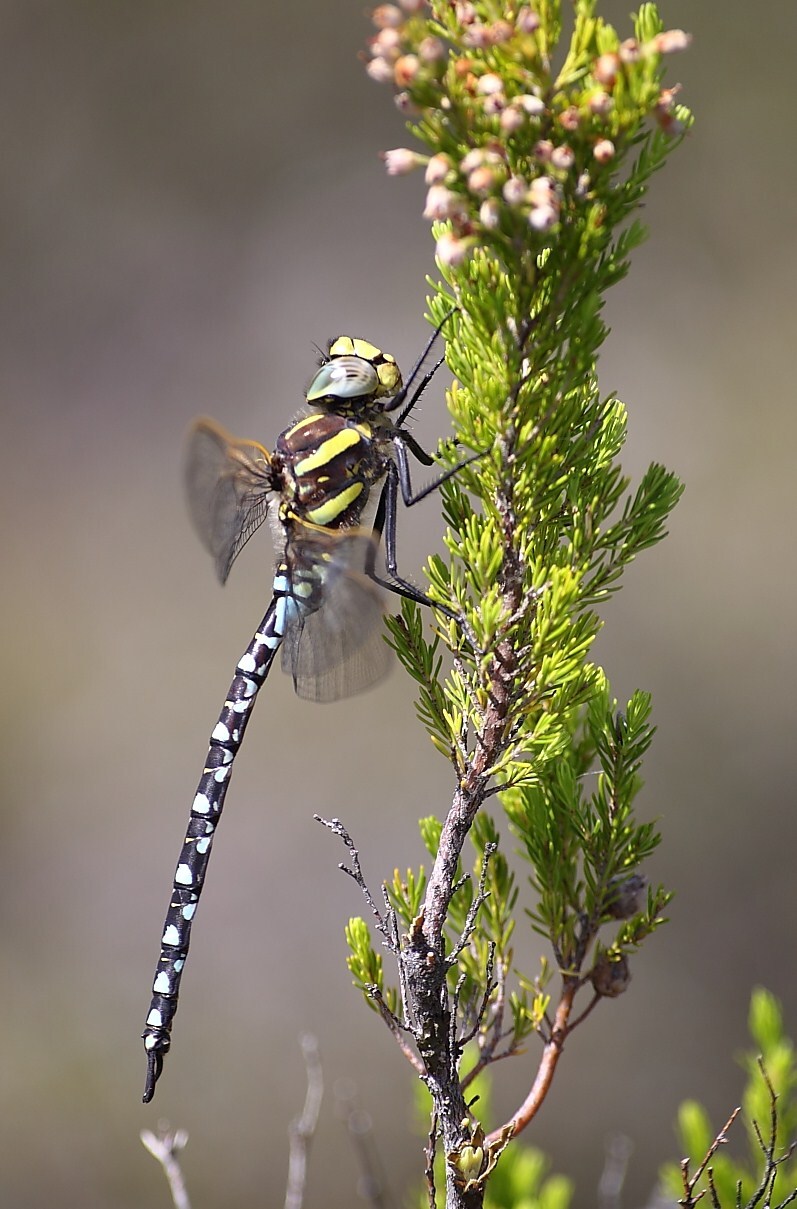
The dragonfly *Aeshnajuncea* (photo by Albano Soares).

**Figure 3a.**
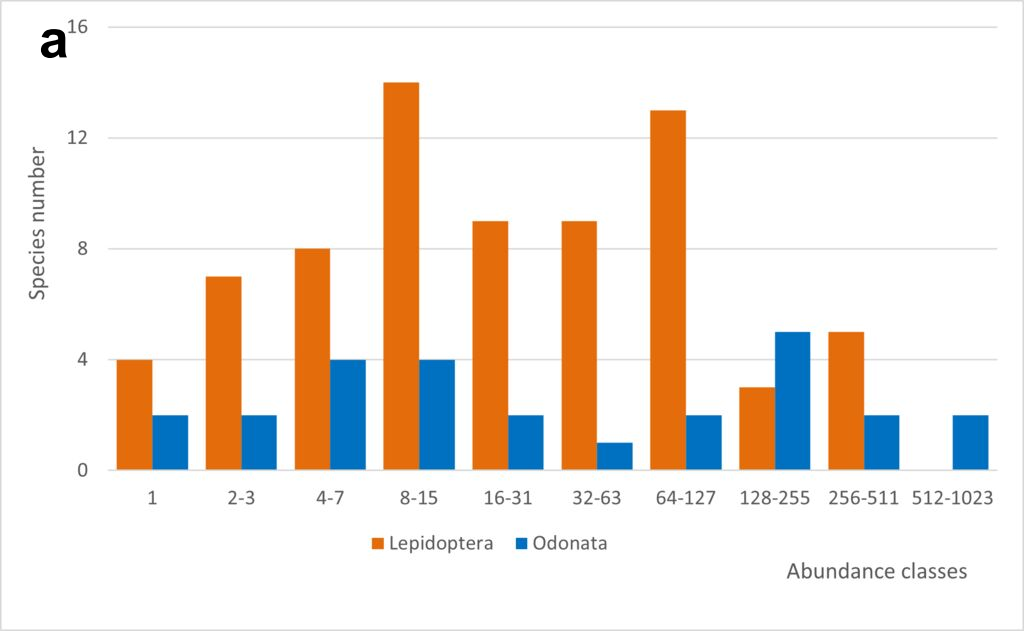
SADs of lepidopterans and odonates. Data were binned in modified log2 abundance classes following Gray et al. (2006).

**Figure 3b.**
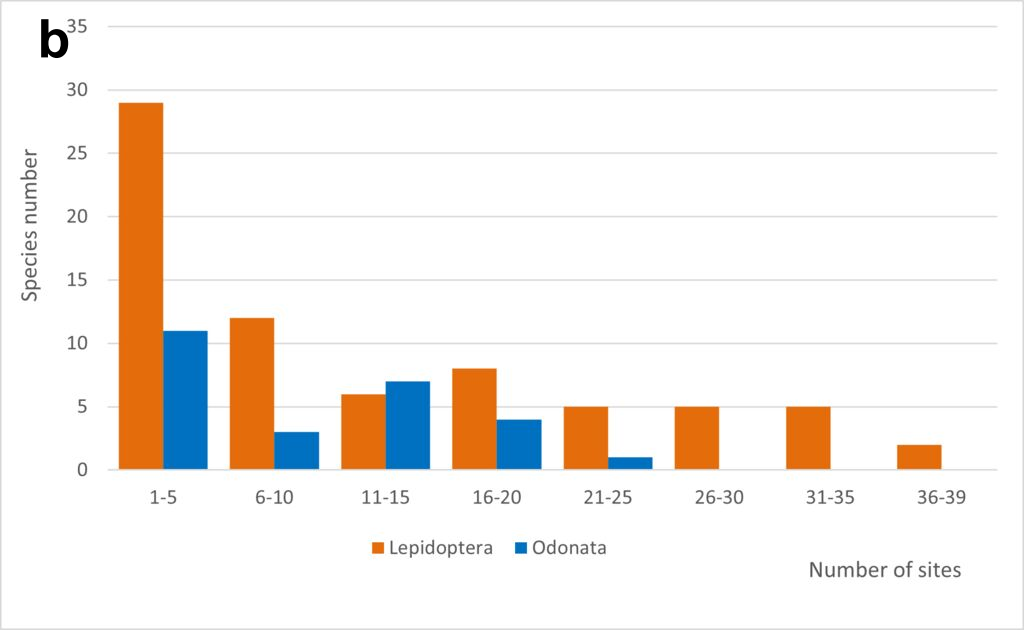
OFDs of lepidopterans and odonates. Data were grouped in site occupancy frequency classes, each comprising 12.5% of the total number of sampling locations.

**Table 1.** List of the study sites with an indication of their location (in decimal degrees WGS84) and habitat-type.

**Site**	**Habitat-type**	**Latitude**	**Longitude**
Conchos	Montane vegetation	-7.61496	40.362301
Corgo das Mós	Montane vegetation	-7.57271	40.401299
Erva da Fome	Montane vegetation	-7.60325	40.391899
Fonte dos Perús	Montane vegetation	-7.62165	40.344002
Lagoacho	Montane vegetation	-7.61437	40.3932
Penha do Gato	Montane vegetation	-7.66434	40.350498
Redonda	Montane vegetation	-7.62864	40.374802
Rodeio Grande	Montane vegetation	-7.64252	40.341202
Seca	Montane vegetation	-7.63228	40.371101
Torre	Montane vegetation	-7.61836	40.317501
Vale das Éguas	Montane vegetation	-7.56813	40.3997
Vale de Perdiz	Montane vegetation	-7.5942	40.4081
Covão do Curral	Mountain lake	-7.63973	40.370899
Covão do Forno	Mountain lake	-7.63604	40.3689
Covão do Meio	Mountain lake	-7.63026	40.333199
Covão do Quelhas	Mountain lake	-7.62676	40.3279
Covão dos Conchos	Mountain lake	-7.60941	40.363701
Lagoa Comprida 1	Mountain lake	-7.64247	40.364101
Lagoa Comprida 2	Mountain lake	-7.62742	40.3591
Lagoa da Francelha	Mountain lake	-7.63331	40.3297
Lagoa do Ângelo	Mountain lake	-7.6327	40.3531
Lagoa Escura	Mountain lake	-7.63796	40.355099
Lagoa Redonda	Mountain lake	-7.6247	40.370201
Lagoa Seca	Mountain lake	-7.6312	40.3713
Lagoa Serrano	Mountain lake	-7.63142	40.3283
Lagoacho das Favas	Mountain lake	-7.63631	40.363602
Lagoacho SE	Mountain lake	-7.62153	40.3801
Lagoacho W	Mountain lake	-7.62385	40.383801
Vale do Rossim NE	Mountain lake	-7.58259	40.399502
Vale do Rossim SW	Mountain lake	-7.59169	40.396301
Cabeça	Mountain stream	-7.71926	40.318501
Loriga	Mountain stream	-7.67507	40.329201
Ponte de Jugais-Ribeira da Caniça	Mountain stream	-7.70396	40.3848
Ponte de Jugais-Rio Alva	Mountain stream	-7.706	40.384602
Porto do Boi	Mountain stream	-7.6756	40.371799
Ribeira da Fervença	Mountain stream	-7.5906	40.4039
Ribeira da Nave	Mountain stream	-7.6337	40.334202
Ribeira da Pragueira	Mountain stream	-7.65328	40.359501
Sabugueiro	Mountain stream	-7.6407	40.4063

**Table 2.** Species abundance and occupancy of lepidopterans from the study sites in Serra da Estrela.

**Family**	**Species/Subspecies**	**Abundance**	**Occupancy**
Hesperiidae	*Hesperiacomma* (Linnaeus, 1758)	132	29
Hesperiidae	*Ochlodessylvanus* (Esper, 1777)	4	3
Hesperiidae	*Pyrgusmalvoides* (Elwes & Edwards, 1897)	15	10
Hesperiidae	*Thymelicusacteon* (Rottemburg, 1775)	3	2
Hesperiidae	*Thymelicuslineola* (Ochsenheimer, 1808)	11	4
Hesperiidae	*Thymelicussylvestris* (Poda, 1761)	44	14
Papilionidae	*Iphiclidesfeisthamelii* (Duponchel, 1832)	11	7
Pieridae	*Anthochariscardamines* (Linnaeus, 1758)	5	1
Pieridae	*Coliascroceus* (Geoffroy, 1785)	256	38
Pieridae	*Gonepteryxrhamni* (Linnaeus, 1758)	61	23
Pieridae	*Leptideasinapis* (Linnaeus, 1758)	69	18
Pieridae	*Pierisbrassicae* (Linnaeus, 1758)	14	9
Pieridae	*Pierisnapi* (Linnaeus, 1758)	58	12
Pieridae	*Pierisrapae* (Linnaeus, 1758)	270	36
Pieridae	*Pontiadaplidice* (Linnaeus, 1758)	71	20
Lycaenidae	*Ariciacramera* Eschscholtz, 1821	59	20
Lycaenidae	*Callophrysrubi* (Linnaeus, 1758)	2	2
Lycaenidae	*Celastrinaargiolus* (Linnaeus, 1758)	107	32
Lycaenidae	*Cyanirissemiargus* (Rottemburg, 1775)	63	9
Lycaenidae	*Glaucopsychemelanops* (Boisduval, 1828)	2	1
Lycaenidae	*Laeosopisroboris* (Esper, 1789)	9	3
Lycaenidae	*Lampidesboeticus* (Linnaeus, 1767)	18	9
Lycaenidae	*Leptotespirithous* (Linnaeus, 1767)	93	25
Lycaenidae	*Lycaenaalciphron* (Rottemburg, 1775)	22	16
Lycaenidae	*Lycaenableusei* Oberthur, 1884	6	2
Lycaenidae	*Lycaenaphlaeas* (Linnaeus, 1761)	81	26
Lycaenidae	*Lycaenatityrus* (Poda, 1761)	3	2
Lycaenidae	*Plebejusargus* (Linnaeus, 1758)	336	31
Lycaenidae	*Polyommatusicarus* (Rottemburg, 1775)	73	20
Lycaenidae	*Satyriumesculi* (Hübner, 1804)	2	1
Lycaenidae	*Satyriumspini* (Denis & Schiffermüller, 1775)	25	5
Nymphalidae	*Aglaisio* (Linnaeus, 1758)	12	9
Nymphalidae	*Aglaisurticae* (Linnaeus, 1758)	24	8
Nymphalidae	*Apaturailia* (Denis & Schiffermüller, 1775)	1	1
Nymphalidae	*Argynnisadippe* (Denis & Schiffermüller, 1775)	61	16
Nymphalidae	*Argynnisaglaja* (Linnaeus, 1758)	6	4
Nymphalidae	*Argynnispandora* (Denis & Schiffermüller, 1775)	179	34
Nymphalidae	*Argynnispaphia* (Linnaeus, 1758)	9	6
Nymphalidae	*Brintesiacirce* (Fabricius, 1775)	115	31
Nymphalidae	*Charaxesjasius* (Linnaeus, 1767)	1	1
Nymphalidae	*Coenonymphadorus* (Esper, 1782)	10	1
Nymphalidae	*Coenonymphaglycerioniphioides* Staudinger, 1870	16	7
Nymphalidae	*Coenonymphapamphilus* (Linnaeus, 1758)	8	7
Nymphalidae	*Euphydryasaurinia* (Rottemburg, 1775)	6	1
Nymphalidae	*Hipparchiafidia* (Linnaeus, 1767)	27	5
Nymphalidae	*Hipparchiahermione* (Linnaeus, 1764)	149	32
Nymphalidae	*Hipparchiasemele* (Linnaeus, 1758)	89	25
Nymphalidae	*Hipparchiastatilinus* (Hufnagel, 1766)	62	15
Nymphalidae	*Hyponephelelycaon* (Rottemburg, 1775)	71	21
Nymphalidae	*Issorialathonia* (Linnaeus, 1758)	106	28
Nymphalidae	*Lasiommatamaera* (Linnaeus, 1758)	15	2
Nymphalidae	*Lasiommatamegera* (Linnaeus, 1767)	63	24
Nymphalidae	*Limenitisreducta* Staudinger, 1901	9	4
Nymphalidae	*Maniolajurtina* (Linnaeus, 1758)	25	7
Nymphalidae	*Melanargialachesis* (Hübner, 1790)	312	26
Nymphalidae	*Melanargiaoccitanica* (Esper, 1793)	3	1
Nymphalidae	*Melanargiarussiae* (Esper, 1783)	11	3
Nymphalidae	*Melitaeadeione* (Geyer, 1832)	21	8
Nymphalidae	*Melitaeanevadensis* Oberthür, 1904	42	12
Nymphalidae	*Melitaeaparthenoides* Keferstein, 1851	7	3
Nymphalidae	*Melitaeaphoebe* (Denis & Schiffermüller, 1775)	7	2
Nymphalidae	*Melitaeatrivia* (Denis & Schiffermüller, 1775)	8	2
Nymphalidae	*Nymphalisantiopa* (Linnaeus, 1758)	13	12
Nymphalidae	*Nymphalispolychloros* (Linnaeus, 1758)	1	1
Nymphalidae	*Parargeaegeria* (Linnaeus, 1758)	90	16
Nymphalidae	*Polygoniac-album* (Linnaeus, 1758)	19	3
Nymphalidae	*Pyroniacecilia* (Vallantin, 1894)	2	2
Nymphalidae	*Pyroniatithonus* (Linnaeus, 1767)	103	15
Nymphalidae	*Satyrusactaea* (Esper, 1781)	268	30
Nymphalidae	*Vanessaatalanta* (Linnaeus, 1758)	4	3
Nymphalidae	*Vanessacardui* (Linnaeus, 1758)	70	19
Nymphalidae	*Vanessavirginiensis* (Drury, 1773)	1	1

**Table 3.** Species abundance and occupancy of odonates from the study sites in Serra da Estrela.

**Family**	**Species**	**Abundance**	**Occupancy**
Calopterygidae	*Calopteryxhaemorrhoidalis* (Vander Linden, 1825)	5	1
Calopterygidae	*Calopteryxvirgo* (Linnaeus, 1758)	203	12
Calopterygidae	*Calopteryxxanthostoma* (Charpentier, 1825)	12	1
Lestidae	*Lestesdryas* Kirby, 1890	374	19
Lestidae	*Lestesvirens* (Charpentier, 1825)	335	15
Lestidae	*Lestesviridis* (Vander Linden, 1825)	5	2
Coenagrionidae	*Ceriagriontenellum* (de Villers, 1789)	1	1
Coenagrionidae	*Enallagmacyathigerum* (Charpentier, 1840)	579	16
Coenagrionidae	*Ischnuragraellsii* (Rambur, 1842)	10	5
Coenagrionidae	*Pyrrosomanymphula* (Sulzer, 1776)	71	13
Platycnemididae	*Platycnemislatipes* Rambur, 1842	12	3
Aeshnidae	*Aeshnacyanea* (Müller, 1764)	26	12
Aeshnidae	*Aeshnajuncea* (Linnaeus, 1758)	183	19
Aeshnidae	*Anaximperator* Leach, 1815	28	12
Aeshnidae	*Boyeriairene* McLachlan, 1896	104	7
Gomphidae	*Onychogomphusuncatus* (Charpentier, 1840)	54	7
Cordulegastridae	*Cordulegasterboltonii* (Donovan, 1807)	182	16
Corduliidae	*Oxygastracurtisii* (Dale, 1834)	2	1
Macromiidae	*Macromiasplendens* (Pictet, 1843)	4	1
Libellulidae	*Libelluladepressa* Linnaeus, 1758	1	1
Libellulidae	*Libellulaquadrimaculata* Linnaeus, 1758	825	22
Libellulidae	*Orthetrumcoerulescens* (Fabricius, 1798)	7	3
Libellulidae	*Sympetrumflaveolum* (Linnaeus, 1758)	190	12
Libellulidae	*Sympetrumfonscolombii* (Selys, 1840)	13	7
Libellulidae	*Sympetrumsanguineum* (Müller, 1764)	140	12
Libellulidae	*Sympetrumstriolatum* (Charpentier, 1840)	2	2
